# Surgical and audiological results of bone-anchored hearing aids: comparison of two surgical techniques

**DOI:** 10.1016/j.bjorl.2020.07.003

**Published:** 2020-08-19

**Authors:** Maria Stella Arantes do Amaral, Francine Raquel dos Santos, Fabiana Danieli, Eduardo T. Massuda, Ana Cláudia Mirândola Barbosa Reis, Miguel Angelo Hyppolito

**Affiliations:** a Universidade de São Paulo, Faculdade de Medicina de Ribeirão Preto, Hospital das Clínicas, Divisão de Otorrinolaringologia, Ribeirão Preto, SP, Brazil; b Hospital das Clínicas de Ribeirão Preto, Ribeirão Preto, SP, Brazil; c Universidade de São Paulo, Faculdade de Medicina de Ribeirão Preto, Departamento de Oftalmologia, Otorrinolaringologia e Cirurgia de Cabeça e Pescoço, Ribeirão Preto, SP, Brazil; d Oticon Medical Brasil, São Paulo, SP, Brazil; e Universidade de São Paulo, Faculdade de Medicina de Ribeirão Preto, Departamento de Ciências da Saúde, Ribeirão Preto, SP, Brazil

**Keywords:** Hearing, Hearing loss, Bone conduction, Bone-anchored hearing system

## Abstract

**Introduction:**

The bone-anchored hearing system has become the most viable treatment option for subjects with conductive or mixed hearing loss, who are unable to benefit from conventional hearing aids or middle ear surgery.

**Objective:**

To compare the surgical and audiological outcomes between the minimally-invasive Ponto surgery and a linear incision with soft tissue preservation techniques in bone-anchored hearing system recipients.

**Methods:**

A retrospective study was carried out from January 2017 to June 2018. Forty-two adult patients eligible for unilateral bone-anchored hearing system surgery with the Ponto system were included in the study. The implant and abutment lengths used varied from 3 to 4 mm and from 6 to 14 mm, according to the bone and skin thickness of the participants, respectively.

**Results:**

Twenty-two surgeries were performed using the minimally invasive Ponto surgery technique (52.4%) and 20 (47.6%) using the linear incision. The mean age of the subjects implanted with minimally invasive Ponto surgery and linear incision techniques were 42.0 and 33.3 years old, respectively. Ten male (45,5%) and 14 (70%) female patients were implanted using minimally invasive Ponto surgery and the linear incision techniques, respectively. There were no differences between pure tone audiometric thresholds and monosyllabic word recognition scores of the subjects, when comparing both surgical techniques. The minimally invasive Ponto surgery technique significantly reduced the surgical time compared to the linear incision technique. There were no differences between both surgical techniques for skin-related complications; (Holgers 3 and 4) which occurred in 18.8% for MIPS and in 25% for linear incision. Subjects included in the minimally invasive Ponto surgery technique group showed a superior cosmetic outcome, with no surgical scar or additional sutures.

**Conclusion:**

The surgical and audiological outcomes were satisfactory and were not correlated to the surgical technique selected in all subjects. When compared to the linear incision, the minimally invasive Ponto surgery technique showed reduced surgical time and superior esthetic outcomes in the postoperative follow-up.

## Introduction

The Bone-Anchored Hearing System (BAHS) is an implantable hearing rehabilitation method that has been used since 1977, when it was introduced by Tjellström and Carlsson. These devices consist of systems that stimulate the inner ear using bone sound transmission, and they comprise a titanium implant and an audio processor, with or without a percutaneous or transcutaneous abutment, capable of decoding sounds and transmitting them directly to the cochlea. These operations have short surgical times and low complication rates as characteristics, and may be indicated for patients with conductive, mixed hearing loss, or unilateral sensorineural hearing loss who do not benefit from air-conduction hearing rehabilitation techniques or through the use of the conventional hearing aid or middle ear reconstructive surgeries.^1^, ^2^, ^3^ The BAHS, through a percutaneous system, consist of three elements: a titanium implant surgically inserted into the mastoid or squamous process of the temporal bone a few millimeters above the temporal line, a support structure implanted through the skin (abutment), connected to the implant through a screw, and a sound processor anchored to this structure. The implant is fixed to the bone through screw threads and is integrated into the skull through an osteointegration process by the growth of bone tissue in contact with the titanium implant surface.^4^ The sound processor is activated 15 to 90 days after the surgery. The audio processor receives the sound, converts it into vibrations and uses the skull as a conductor to transmit it directly to the cochlea, avoiding transmission by air and thus excluding the external auditory canal and the middle ear.^5^, ^6^, ^7^ The adaptation of the audio processor, in this case, is carried out through direct connection, via percutaneous abutment.^8^

Aiming to improve results and avoid complications, both the implant design and the surgical technique have improved. In the beginning, the subcutaneous tissue of the skin around the abutment and hair follicles was removed. Subsequently, the linear incision technique without soft tissue reduction was introduced.^9^

In 2011, Hultcrantz et al.^9^ described a minimally invasive surgical procedure for the Ponto™ brand bone-anchored hearing aid, manufactured by Oticon Medical, in which a 5-mm dermal puncture was performed to remove the soft tissue required to accommodate the abutment. This technique is known as the MIPS (Minimally Invasive Ponto Surgery) technique.

The literature describes as complications of bone-anchored hearing aids: bleeding, wound dehiscence (spontaneous opening of the surgical stitches or scar along the surgical incision line), persistent pain, flap necrosis, osteointegration failure (which can lead to implant loss), skin trauma and complications, with these being the most frequent ones reported by the authors, with rates varying from 15% to 48.5% of cases.^4^, ^7^ In Brazil, few studies evaluated the influence of the surgical technique on surgical results and their potential complications. To offer more accurate information regarding the available surgical techniques to perform the surgical implantation of bone-anchored hearing aids and the possible complications inherent to them, this study aimed to verify the surgical and audiological results and study the surgical and skin complications with the two techniques (MIPS and linear incision), both with preservation of soft tissues.

## Methods

A retrospective study was carried out of patients submitted to surgery to place bone-anchored hearing aids, from January 2017 to June 2018, in a Hearing Health service in the state of São Paulo. The employed surgical technique was randomly chosen.

The study was approved by the institutional Research Ethics Committee under protocol n. 01508318.5.0000.5440.

We included individuals with a unilateral implant and excluded those who underwent more than one procedure in the same surgical performance, patients who underwent implantation of bone-anchored hearing aids bilaterally and those with bone or dermatological pathologies that could cause osteointegration difficulties or influence surgical wound healing.

The data obtained in the collection involved information about: age, gender, implanted ear, etiology, audiological characteristics of each patient in the pre- and postoperative period (auditory threshold in the sound field and speech perception test), surgical technique used, surgery duration, and surgical and skin complications observed for at least six months of follow-up after the surgery.

To assess skin complications, the classification proposed by Holgers et al. was used^10^, ^11^: Grade 0 = no adverse reactions; Grade 1 = skin with erythema; Grade 2 = skin with erythema and secretion; Grade 3 = granulation tissue; and Grade 4 = inflammation or infection that resulted in the removal of the abutment. Skin complications were divided into minor (Grade 1 and 2) and major (Grade 3 and 4) complications.^12^

The collected audiometric data were related to audiometry performed in the free field, through the means of the 500, 1000, 2000 and 4000 Hz frequencies and compared in the preoperative period and after three months of the postoperative period. For the analysis of speech perception, the test of monosyllable words proposed by Lacerda was applied.^13^ To control the variable related to the equipment itself, in the two studied groups, only Ponto brand implants (Oticon Medical™) with abutments were used.

### Statistical analysis

Descriptive results were presented as mean and standard deviation, or median and Interquartile Range (IQR) in situations where the distribution was asymmetric. The Chi-Square test was used to compare gender and Student's *t* test to compare age between the groups. The Mann–Whitney test was used in the other comparisons between the groups. The comparisons between pre- and postoperative times were obtained using the Wilcoxon Rank Sum Test. The proportion test was applied to test Holgers score between the groups. The Software R, version 3.5.2, was used for all analyses. The level of significance was set at 5%.

## Results

Forty-two unilateral bone-anchored hearing aids surgeries were performed from January 2017 to June 2018, with the MIPS surgical technique being applied to 22 individuals (52.4%) and the linear incision in 20 (47.6%).

The minimum, maximum and mean age at surgery is shown in Table 1, for the two surgical techniques. There was no difference between groups regarding age (*p* = 0.14).

**Table 1:** Demographic and surgical characteristics of the individuals (*n* = 42).

Demographic characteristics		MIPS (*n* = 22)	Linear incision (*n* = 20)
Age (years)	Minimum age	15	7
	Maximum age	71	67
	Mean age	42	33.3
	SD	16	20.9
Gender	Female	14 (63.7%)	10 (50%)
	Male	8 (36.3%)	10 (50%)
Hearing loss etiology	EAC stenosis	6 (27.3%)	1 (5%)
	Microtia	2 (9%)	7 (35%)
	PO otological surgery	7 (31.8%)	8 (40%)
	NS hearing loss	5 (22.8%)	2(10%)
	Syndromic hearing loss	1 (4.5%)	1(5%)
	Otosclerosis	1 (4.5%)	1(5%)

Surgical characteristics		MIPS (*n* = 22)	Linear incision (*n* = 20)
Implanted ear	Right	10 (45.5%)	10 (50%)
	Left	12 (54.5%)	10 (50%)
Surgery duration (min)	Minimum	8	20
	Maximum	40	75
	Mean	21.8	32
	Median	20	27

SD, standard deviation; EAC, external auditory canal; PO otologic surgery, postoperative otologic surgery.

Ten surgeries were performed in female subjects using the MIPS technique and 14 using a linear incision. There was no difference between groups regarding gender (*p* = 0.56). Ten surgeries were also performed in the right ear and 10 in the left ear using the MIPS technique, and 10 in the right ear and 12 in the left ear using the linear incision, with no statistical difference regarding the implanted ear (*p* = 1.00) (Table 1).

The etiologies that led to the need for surgery are also shown in Table 1. It was found that the most frequent etiology was related to postoperative otological surgery events that prevented the patient from using air amplification, occurring in 31.8% of individuals with the MIPS technique and 40% with the linear incision technique.

The audiometric results in the pre- and postoperative phases are shown in Table 2. The data were obtained through sound field audiometry with the mean of the frequencies of 500, 1000, 2000 and 4000 Hz and in the pre-surgical phases without the device and after surgery with the bone-anchored hearing aid, during the adjustment period and the individual's scheduled customary use. There was no difference in the means of hearing thresholds between the different surgical techniques at any time (pre- and postoperative periods). However, there was a significant improvement in hearing for both of them in the postoperative period, as early as at the activation of the audio processor (Table 2).

**Table 2:** Mean of the individuals’ hearing thresholds, in the pre- and postoperative phases, for the two used techniques (*n* = 42).

Surgical technique	Preoperative	Postoperative	*p-*value^a^
	Mean and SD	Mean and SD	
	(dBSPL)	(dBSPL)	
Linear incision	63.6 ± 17.6	27.9 ± 11.1	<0.0001
MIPS	70.3 ± 21.3	30.9 ± 7.4	
*p*-value^b^	0.3	0.41	

SD, standard deviation.

a Comparison between times.

b Comparison between groups.

The speech perception tests in the pre- and postoperative phases are shown in Table 3, for both techniques. It was possible to detect an improvement in the speech perception test (SPT) results for both groups, when the two phases were compared (*p* = 0.0011 and *p* = 0.004). No difference was verified when comparing the two surgical techniques in the pre- and postoperative phases (*p* = 0.86 and 0.81, respectively).

**Table 3:** Results of speech perception tests in the pre- and postoperative phases, with the two employed surgical techniques (*n* = 42).

Surgical technique	*n*	Speech perception test (SPT)		*p*^a^-value
		Preoperative	Postoperative	
Linear incision	20	60.0 (22.0–70.0)	96.0 (92.0–100.0)	0.0011
MIPS	22	48.0 (8.0–64.0)	96.0 (89.0–100.0)	0.004
*p*-value^b^		0.86	0.81	

SPT, speech perception tests for monosyllable words.

a Comparison between times.

b Comparison between groups.

The duration of the surgical procedure for each technique is shown in Table 4. There was a significant difference between the groups regarding the surgical time (*p* = 0.0078), being shorter with the MIPS technique.

**Table 4:** Distribution of individuals in relation to the surgical time in minutes per surgical technique (*n* = 42).

Surgical technique	*n*	Surgical time (min)			
		Minimum	Maximum	Mean	Median
Linear incision	20	20	75	32.0	27.0
MIPS	22	8	40	21.8	20.0

Regarding the surgical complications, we found that skin complications (Holgers ≥ 1)^10^, ^11^ were present in both surgical techniques, that is, in the linear incision technique in 9 of 20 individuals undergoing surgery (45%) and in the MIPS technique, in 10 individuals of 22 submitted to surgery (45%). In addition to these skin complications that occurred in up to 6 months of postoperative follow-up (which were resolved in their entirety), we found a description of intraoperative bleeding in 1 patient undergoing each of the surgical techniques, that is, 5% in the linear incision technique and 4.5% in the MIPS technique. However, as these bleeding episodes were of minor intensity, they were not considered in the list of complications. One case of trauma followed by prosthesis extrusion was also observed in a patient submitted to the linear incision surgery, which was also not considered a complication, as it is not related to the employed surgical technique.

Postoperative cutaneous complications are shown in Table 5, separated into minor complications (Holgers 1 and 2) and major complications (Holgers 3 and 4), using the classification proposed by Holgers et al.^11^

**Table 5:** Distribution of skin complications, according to the Holgers classification, for the two employed surgical techniques (*n* = 42).

Holgers classification	Surgical technique		Chi-square test = 0.026
	MIPS (*n* = 22)	Linear incision (*n* = 20)	
Minor complications (Holgers 1 and 2)	6 (27.7%)	4 (20.0%)	*p* = 0.8275
Major complications (Holgers 3 and 4)	4 (18.18%)	5 (25.0%)	

There was no difference when comparing the two techniques in relation to skin complications (*p* = 0.8275).

## Discussion

The studied groups, differentiated by the employed surgical techniques (MIPS and linear incision), were homogenous in terms of age, gender and operated ear; therefore, these variables did not interfere with the results of the present study. This homogeneity of the sample groups was also shown by Steehler et al.,^14^ who studied 90 individuals submitted to bone-anchored hearing aid surgery and compared five surgical techniques used to implant these devices.

We observed that the mean age was that of adult patients in both groups, considering the limitations for the indication of bone-anchored hearing aid surgery in children. For the insertion of the titanium implant, a minimum thickness of the skullcap of ≥3 mm is necessary.^15^ In Brazil, Ministerial Ordinance N. 2776/GM/MS, of December 18, 2014, indicates the surgery in children over the age of five.^16^

The results of the present study corroborate those of the literature, considering the etiologies that led to the need for the indication of the bone-anchored hearing aid. Calon et al.^4^ reported acquired conductive hearing loss as the most frequent etiology, present in 78.8% for the MIPS and 83.3% for the linear incision technique. This higher percentage may be due to the fact that the etiologies in their study were divided into only three groups: acquired conductive, unilateral hearing loss and congenital conductive hearing loss. Moreover, among the etiologies that led to the need for surgery, only seven had sensorineural hearing loss as an indication, which may reflect a characteristic of the public services in Brazil, considering the specific indications determined by the Brazilian Ministry of Health. The remaining 35 etiologies were due to changes in the middle and outer ear, similar to most clinical studies in which the majority of cases comprise the conductive-type hearing loss as an indication for bone-anchored hearing aid use.^14^

A significant audiological improvement was observed when comparing the pre- and the postoperative periods (Table 2, Table 3), both for the mean of the auditory thresholds and for the speech perception tests, thus confirming the surgical indication for bone-anchored hearing aids in the two studied groups, in agreement with the relevant literature.^2^, ^7^

As in the clinical studies by Calon et al.^4^ and Den Besten et al.,^6^ the surgical time was different in the two groups, being shorter in the individuals undergoing the MIPS technique than in those submitted to the linear incision technique, considered as one of the advantages of MIPS, taking into account the adverse effects caused by the increased time of exposure to anesthetics.^17^, ^18^ Other authors also point out that shorter surgical times lead to lower expenses for health services. This study showed that because the MIPS technique is less invasive, the procedure can be performed on an outpatient basis, costing $450 less per performed procedure.^19^

Intraoperative bleeding, which in our study was not considered a complication, since it was of low intensity, was present in 5% of surgery cases using the linear incision technique and 4.5% using the MIPS technique, data compatible with the authors who showed 3% for MIPS and 3.5% for the linear incision.^5^

Skin complications were the most frequent ones with both surgical techniques, with no differences being observed between the techniques, in agreement with studies in the literature.^7^ There was also no difference when the groups were divided into minor complications (Holgers 0–2) and major complications (Holgers 3–4), data that are in agreement with the literature.^11^

The literature also points out that in the MIPS technique, the incision is smaller and does not require stitches on the skin and, thus, it shows better esthetic outcome in the postoperative period, which could be demonstrated with the application of questionnaires; however, this variable (esthetics) was not the subject of the present study.^20^

## Conclusion

The MIPS surgical technique involves a shorter surgical time than the linear incision technique. There was no significant difference regarding postoperative complications between MIPS and the linear incision surgical techniques. The audiological result for both techniques was similar.

## Conflicts of interest

F.D. works in clinical support for the Oticon Medical company; however, the brand and model of the device to be implanted is acquired by the public service where the collection took place, through a bidding process, without the participation of the professionals and team leaders involved with these surgeries. The authors declare no conflicts of interest.
